# Intravenous immunoglobulin-based adjuvant therapy for severe COVID-19: a single-center retrospective cohort study

**DOI:** 10.1186/s12985-021-01575-3

**Published:** 2021-05-21

**Authors:** Xiao Hou, Li Tian, Lei Zhou, Xinhua Jia, Li Kong, Yitao Xue, Hao Hao, Xianqing Meng, Feihu Zhang, Xiaobin Dong

**Affiliations:** 1 Pelvic Floor Disease Center, Shandong Provincial Maternal and Child Health Care Hospital, Jinan, Shandong People’s Republic of China; 2 Department of Lung Disease, Affiliated Hospital of Shandong University of Traditional Chinese Medicine, Jinan, Shandong People’s Republic of China; 3 Department of Pulmonary Critical Care Medicine, The First Affiliated Hospital of Shandong First Medical University, Jinan, Shandong People’s Republic of China; 4 Department of Critical Care Medicine, Affiliated Hospital of Shandong University of Traditional Chinese Medicine, Jinan, Shandong People’s Republic of China; 5 Department of Cardiovascular Diseases, Affiliated Hospital of Shandong University of Traditional Chinese Medicine, Jinan, Shandong People’s Republic of China

**Keywords:** SARS-CoV-2, COVID-19, IVIG, Mortality, Mechanical ventilation, Hospital length of stay

## Abstract

**Objective:**

Coronavirus disease 2019 (COVID-19) is a major challenge facing the world. Certain guidelines issued by National Health Commission of the People's Repubilic of China recommend intravenous immunoglobulin (IVIG) for adjuvant treatment of COVID-19. However, there is a lack of clinical evidence to support the use of IVIG.

**Methods:**

This single-center retrospective cohort study included all adult patients with laboratory-confirmed severe COVID-19 in the Respiratory and Critical Care Unit of Dabie Mountain Regional Medical Center, China. Patient information, including demographic data, laboratory indicators, the use of glucocorticoids and IVIG, hospital mortality, the application of mechanical ventilation, and the length of hospital stay was collected. The primary outcome was the composite end point, including death and the use of mechanical ventilation. The secondary outcome was the length of hospital stay.

**Results:**

Of the 285 patients with confirmed COVID-19, 113 severely ill patients were included in this study. Compared to the non-IVIG group, more patients in the IVIG group reached the composite end point [12 (25.5%) vs 5 (7.6%), *P* = 0.008] and had longer hospital stay periods [23.0 (19.0–31.0) vs 16.0 (13.8–22.0), *P* < 0.001]. After adjusting for confounding factors, differences in primary outcomes between the two groups were not statistically significant (*P* = 0.167), however, patients in the IVIG group had longer hospital stay periods (*P* = 0.041).

**Conclusion:**

Adjuvant therapy with IVIG did not improve in-hospital mortality rates or the need for mechanical ventilation in severe COVID-19 patients. Our study does not support the use of immunoglobulin in patients with severe COVID-19 patients.

## Introduction

Coronavirus disease 2019 (COVID-19) is a prevalent respiratory disease that is caused by severe acute respiratory syndrome coronavirus 2 (SARS-CoV-2). Although most COVID-19 patients exhibit mild symptoms, approximately 15% of COVID-19 patients progress to severe pneumonia while 5% develop acute respiratory distress syndrome [1, 2]. Clinical studies are evaluating the efficacies of antiviral drugs for the treatment of COVID-19 [3, 4].

COVID-19 is associated with various inflammatory responses. As the disease progresses, levels of systemic proinflammatory cytokines and biomarkers increase, and are correlated with poor prognosis, indicating that the inflammatory storm plays an important role in disease progression. Immune response regulation may prevent the occurrence of organ dysfunction. Intravenous immunoglobulin (IVIG) is a non-specific immunomodulator. Continuous IVIG infusion elevates plasma IgG levels and effectively neutralizes respiratory pathogens, thereby promoting disease recovery and shortening disease course. IVIG has been shown to improve the body's defense system, block related receptors in target cells, and prevent pathogens from further damaging target cells [5]. In addition, IVIG affects lymphocytes differentiation and maturation, blocks normal leukocytes immune responses, inhibits cytokine production, and suppresses inflammatory injury [6–8]. Based on the clinical efficacy of IVIG on other viral diseases, it has been postulated that IVIG is beneficial for COVID-19 patients [6, 9, 10]. Although trial version guidelines issued by the National Health Commission and Administration of Traditional Chinese Medicine propose that IVIG can be considered for severely and critically ill COVID-19 patients, the role of IVIG in the treatment of COVID-19 has not been established. Therefore, we retrospectively determined the correlation between the application of IVIG adjuvant therapy and the prognosis of severe COVID-19 patients.

## Methods

### Research design and subjects

This single-center retrospective cohort study was conducted in the Respiratory and Critical Care Unit of Dabie Mountain Regional Medical Center under the jurisdiction of the Shandong Medical Team. Dabie Mountain Regional Medical Center is a designated hospital in Huanggang City (Hubei Province, China) for the treatment of patients with confirmed COVID-19. All patients were transferred from other hospitals, and laboratory confirmation was performed by their local health bureau in accordance with the diagnostic standards of the National Health Commission [11]. We retrospectively analyzed patients with severe COVID-19 newly admitted between January 28 and February 25, 2020. According to the World Health Organization (WHO) interim guidance [12], severe COVID-19 was defined as having one of the following three conditions: respiratory rate ≥ 30 breaths/min, severe respiratory distress, or peripheral capillary oxygen saturation (SPO2) ≤ 93% when inhaling room air. The identification of critically ill patients was achieved by reviewing all available electronic data such as admission records, nursing records, and treatment logs.

### Inclusion and exclusion criteria

All adult patients with severe COVID-19 were screened. The exclusion criteria for the study included age < 18 years, unstable tumor or blood disease, pregnancy, multiple injuries, craniocerebral trauma, mechanical ventilation on admission, and lack of important data.

### Data collection

We collected the patients’ information, including age, sex, concomitant diseases (including hypertension, diabetes, coronary heart disease, chronic obstructive pulmonary disease (COPD), and cerebrovascular disease), the time from onset to admission, the highest body temperature before admission, laboratory examination indicators at admission (including white blood cell count, lymphocyte count, platelet count, alanine aminotransferase (ALT), aspartate aminotransferase (AST), serum creatinine, prothrombin time (PT), and d-dimer), the use of glucocorticoids (including methylprednisone, dexamethasone and hydrocortisone), and the use of intravenous immunoglobulin (IVIG). The prognostic indicators of the patients were also collected, including hospital mortality, the use of mechanical ventilation and the length of hospital stay. All data were collected by two investigators (Hao Hao and Meng Xianqing) separately. If there was any deviation in agreement, a third investigator (Kong Li) made the final decision. All missing continuous variables were replaced by the median or mean. The primary prognostic endpoint was the composite end point, including death or the use of mechanical ventilation. The secondary prognostic endpoint was the length of hospital stay.

### Statistical analysis

Statistical analysis was performed using SPSS 26.0. The data that fit a normal distribution are expressed as the mean ± standard deviation (x ± s), and comparisons between groups was performed using the t test. Non-normally distributed data are expressed as the median (quartile) (M (Q)) and were compared using a nonparametric test. Count data are expressed as the frequency and percentage, and comparisons between groups were performed using the chi-squared test. The correlation between IVIG and prognosis was examined using logistic regression analysis (the results are presented as the OR value and 95% C.I.). The correlation between IVIG and the length of hospital stay was examined using multivariate linear regression analysis. Two-tailed *P* < 0.05 indicated a statistically significant difference.

## Results

**Fig. 1:**
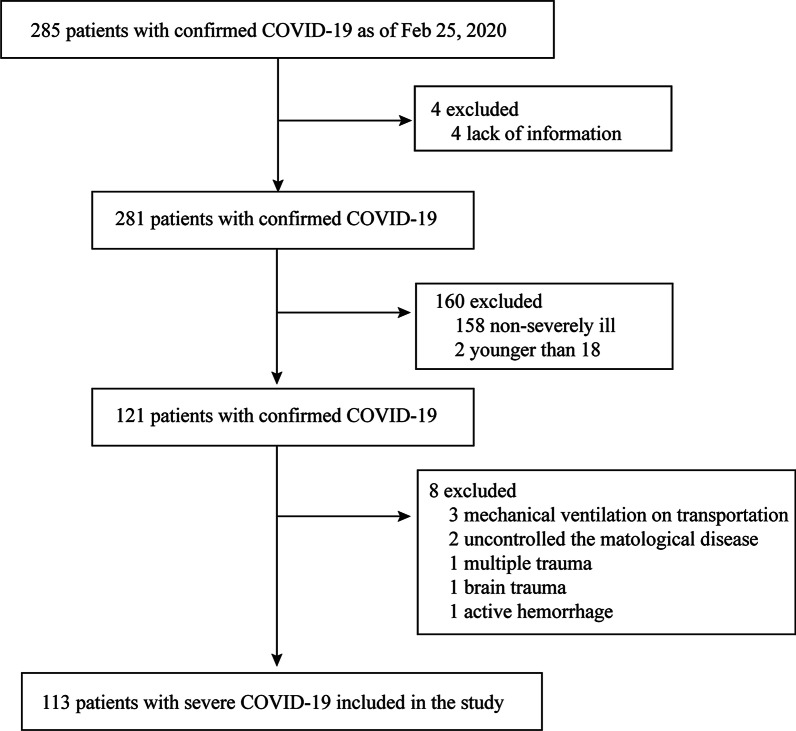
The research flow chart

From January 28 to February 25, 2020, a total of 285 patients with confirmed COVID-19 were treated in the Respiratory and Critical Care Unit of Dabie Mountain Regional Medical Center under the jurisdiction of the Shandong Medical Team. Among these patients, 113 (39.6%) were included in the present study. The research flow chart is shown in Fig. 1.

The comparison of baseline data is shown in Table 1. The age of the patients was 55.1 ± 14.2 years, and 52 (46%) patients were female. Among these patients, 39 (34.5%) suffered from comorbidities, including 26 (23.0%) with hypertension, 13 (11.5%) with diabetes, 7 (6.2%) with coronary heart disease, 6 (5.3%) with COPD, and 4 (3.5%) with cerebrovascular diseases. The maximum body temperature before admission was 38.5 °C (37.7–39.0), and the time from onset to hospitalization was 7.0 days (5.0–10.0). Forty-seven patients (41.6%) received IVIG. Compared with patients who did not receive IVIG, fewer patients who received IVIG therapy had coronary heart disease [0 (0) vs 7 (10.6%), *P* = 0.021]. In addition, patients who received IVIG therapy had a higher body temperature [38.9 (38.2–39.0) vs 38.0 (37.5–38.8), *P* = 0.002] before hospital admission, a higher white blood cell count [7.45 (4.73–9.42) vs 5.00 (3.68–6.79), *P* < 0.001], a lower lymphocyte count [0.79 (0.62–1.21) vs 1.24 (0.90–1.75), *P* < 0.001], and a higher glutamate aminotransferase level [29.7 (18.0–47) vs 21.0 (14.2–29.2), *P* = 0.008]. More patients in the IVIG group used glucocorticoids [39 (83.0%) vs 18 (27.3%), *P* < 0.001]. In contrast, there were no statistically significant differences between the groups of patients in age, sex, comorbidities, concurrent hypertension, diabetes, COPD, cerebrovascular disease, platelet count, ALT level, serum creatinine level, PT level and d-dimer level.

**Table 1:** Demographic data and clinical characteristics of patients in the IVIG and non-IVIG groups

Parameter	Total n = 113	Non-IVIG n = 66	IVIG n = 47	*P*
Age	55.1 ± 14.2	55.3 ± 15.5	54.8 ± 12.4	0.865
Female	52 (46.0%)	32 (48.5%)	20 (42.6%)	0.533
Comorbidity	39 (34.5%)	23 (34.8%)	16 (34.0%)	0.929
Hypertension	26 (23.0%)	14 (21.2%)	12 (25.5%)	0.591
Diabetes	13 (11.5%)	6 (9.1%)	7 (14.9%)	0.341
Coronary heart disease	7 (6.2%)	7 (10.6%)	0 (0)	0.021
COPD	6 (5.3%)	5 (7.6%)	1 (2.1%)	0.203
Cerebrovascular disease	4 (3.5%)	4 (6.1%)	0 (0)	0.086
Onset time	7.0 (5.0–10.0)	7.0 (5.0–9.2)	7.0 (5.0–10.0)	0.268
Maximum body temperature	38.5 (37.8–39.0)	38.0 (37.5–38.8)	38.9 (38.2–39.0)	0.002
WBC	5.61 (4.09–8.04)	5.00 (3.68–6.79)	7.45 (4.73–9.42)	< 0.001
LYM	1.07 (0.72–1.51)	1.24 (0.90–1.75)	0.79 (0.62–1.21)	< 0.001
PLT	200.0 (153.0–256.0)	203.0 (149.8–252.2)	190.0 (159.0–257.0)	0.836
ALT	23.0 (16.5–37.2)	21.0 (14.2–29.2)	29.7 (18.0–47)	0.008
AST	21.0 (17.0–33.0)	20.6 (16.8–26.2)	22.0 (17.0–40.0)	0.287
Cr	78.1 ± 23.9	78.2 ± 24.3	78.8 ± 23.6	0.896
PT	44.6 ± 1.3	11.4 ± 1.2	11.9 ± 1.4	0.56
DD	192.0 (115.0–456.0)	182.5 (105.8–294.5)	216.0 (125.0–840.0)	0.249
GC	57 (50.4%)	18 (27.3%)	39 (83.0%)	< 0.001

*IVIG* intravenous immunoglobulin, *COPD* chronic obstructive pulmonary disease, *WBC* white blood cell, *LYM* lymphocyte, *PLT* platelet, *ALT* alanine aminotransferase, *AST* aspartate aminotransferase, *Cr* serum creatinine, *PT* prothrombin time, *DD* d-dimer, *GC* Glucocorticoid

The outcome indicators are shown in Table 2. A total of 17 (15.0%) patients reached the composite end point, including 13 (11.5%) patients who died and 11 (9.7%) patients who received mechanical ventilation. Compared with the non-IVIG group, more patients in the IVIG group reached the composite end point [12 (25.5%) vs 5 (7.6%), *P* = 0.008]. Specifically, an increased percentage of patients in the IVIG group died [9 (19.1%) vs 4 (6.1%), *P* = 0.032] or received mechanical ventilation [10 (21.3%) vs 1 (1.5%), *P* < 0.001].

**Table 2:** The effect of IVIG therapy on the primary and secondary prognostic endpoints

Parameter	Total n = 113	Non-IVIG n = 66	IVIG n = 47	*P*	P*
Composite end point	17 (15.0%)	5 (7.6%)	12 (25.5%)	0.008	0.167
Death	13 (11.5%)	4 (6.1%)	9 (19.1%)	0.032	–
Mechanical ventilation	11 (9.7%)	1 (1.5%)	10 (21.3%)	< 0.001	–
Length of hospital stay	20.0 (15.0–25.0)	16.0 (13.8–22.0)	23.0 (19.0–31.0)	< 0.001	0.041

^*^Corrected by age, sex, comorbidities, maximum body temperature, WBC, LYM, AST, PT, DD and use of glucocorticoids

The univariate analysis results are provided in Table 3. Several variables were statistically significant differences between patients who reached the prognostic endpoint and those who did not reach the prognostic endpoint; those variables were age [59.0 (52.0–73.5) vs 54.0 (45.2–62.0), *P* = 0.042], maximum body temperature [38.9 (38.2–39.4) vs 38.4 (37.6–39.0), *P* = 0.041], female [4 (23.5%) vs 48 (50.0%), *P* = 0.044], comorbidities [11 (64.7%) vs 28 (29.2%), *P* = 0.004], white blood cell count [8.43 (6.58–16.21) vs 5.28 (4.02–7.22), *P* = 0.001], lymphocyte count [36.0 (18.0–45.4) vs 20.6 (17.0–27.0), *P* = 0.029], AST level [36.0 (18.0–45.4) vs 20.6 (17.0–27.0), *P* = 0.029], PT (12.9 ± 1.6 vs 11.4 ± 1.2, *P* < 0.001), d-dimer level [851.0 (217.0–9954.5) vs 172.0 (107.9–285.5), *P* < 0.001], use of glucocorticoids [14 (82.4%) vs 43 (44.8%), *P* = 0.004], and use of IVIG [12 (70.6%) vs 35 (36.5%), *P* = 0.008]. After adjusting for the above confounding factors, there was no statistically significant difference in the rate of reaching the composite end point between the IVIG group and the non-IVIG group (OR = 2.605, 95%CI 0.67–10.10, *P* = 0.167). Multivariate logistic regression analysis showed that the factors related to reaching the prognostic endpoint included comorbidities (OR = 4.187, 95%CI 1.14–15.41, *P* = 0.031), white blood cell count (OR = 1.18, 95%CI 1.06–1.31, *P* = 0.003), and PT (OR = 2.15, 95%CI 1.30–3.54, *P* = 0.003).

**Table 3:** Univariate analysis of the patients who reached the composite end point and the patients who failed to reach the composite end point

Parameter	Patients who reached the composite end point n = 96	Patients who failed to reach the composite end point n = 17	*P*
Age	54.0 (45.2–62.0)	59.0 (52.0–73.5)	0.042
Female	48 (50.0%)	4 (23.5%)	0.044
Comorbidity	28 (29.2%)	11 (64.7%)	0.004
Hypertension	19 (19.8%)	7 (41.2%)	0.053
Diabetes	9 (9.4%)	4 (23.5%)	0.092
Coronary heart disease	4 (4.2%)	3 (17.6%)	0.034
COPD	4 (4.2%)	2 (11.8%)	0.198
Cerebrovascular disease	2 (2.1%)	2 (11.8%)	0.046
Time of onset	7.0 (5.0–10.0)	7.0 (6.5–10.0)	0.499
Body temperature	38.4 (37.6–39.0)	38.9 (38.2–39.4)	0.041
WBC	5.28 (4.02–7.22)	8.43 (6.58–16.21)	0.001
LYM	1.12 (0.78–1.54)	0.67 (0.55–1.03)	0.001
PLT	201.0 (159.2–258.5)	191.0 (124.0–213.5)	0.086
ALT	23.0 (15.0–34.7)	29.7 (21.5–74.0)	0.065
AST	20.6 (17.0–27.0)	36.0 (18.0–45.4)	0.029
Cr	76.6 ± 22.2	88.6 ± 30.5	0.056
PT	11.4 ± 1.2	12.9 ± 1.6	< 0.001
DD	172.0 (107.9–285.5)	851.0 (217.0–9954.5)	< 0.001
GC	43 (44.8%)	14 (82.4%)	0.004
IVIG	35 (36.5%)	12 (70.6%)	0.008

*IVIG* intravenous immunoglobulin, *COPD* chronic obstructive pulmonary disease, *WBC* white blood cell, *LYM* lymphocyte, *PLT* platelet, *ALT* alanine aminotransferase, *AST* aspartate aminotransferase, *Cr* serum creatinine, *PT* prothrombin time, *DD* d-dimer, *GC* Glucocorticoid

Analysis of the secondary prognostic endpoints showed that the length of hospital stay was 20.0 days (15.0–25.0). Compared with the non-IVIG group, patients in the IVIG group had a longer hospital stay [23.0 days (19.0–31.0) vs 16.0 (13.8–22.0), *P* < 0.001]. After adjusting for confounding factors including age, sex, maximum body temperature, comorbidities, AST, hormones, WBC, d-dimer and PT, it was found that the patients in the IVIG group had a significantly longer hospital stay compared with that for the patients in the non-IVIG group (*P* = 0.041) (Table 2).

## Discussion

This study explored the correlation between the application of IVIG and the prognosis of patients with severe COVID-19. The results showed that 41.6% of critically ill patients received IVIG therapy. Approximately 25.5% of the patients in the IVIG group reached the composite end point, a percentage greater than that in the non-IVIG group. However, multivariate logistic analysis showed that the use of IVIG was not correlated with the poor prognosis of patients with severe COVID-19. The high mortality rate in the IVIG group might be related to the lower lymphocyte count, higher white blood cell count and maximum body temperature. According to the results of previous studies [13–15], these factors were all related to the poor prognosis of COVID-19 patients, suggesting that the condition of the patients in the IVIG group was more severe.

Although certain interim guidelines recommend IVIG adjuvant therapy for patients with severe COVID-19, there is a lack of effective evidence-based proof supporting this treatment [11]. Xie et al. [16] retrospectively studied 58 patients with severe or critical COVID-19, all of whom were treated with IVIG. The study found that the administration of IVIG within 48 h was related to a reduction in 28-day mortality, length of hospital stay and time in the ICU. In the study conducted by Xie et al., the overall 28-day mortality rate was 39.6%, which was much higher than the mortality rate in our study (11.5%). Such phenomenon indicates that the condition of the patients included in Xie’s study was more severe. Shao et al. [17] conducted a multicenter retrospective cohort study that included 325 patients with confirmed severe or critical COVID-19. Among the 325 patients, 222 (68%) had severe COVID-19, and 103 (32%) had critical COVID-19. No significant differences were found in the 28-day mortality rate and 60-day mortality rate between the IVIG group and the non-IVIG group. After adjusting for baseline data such as age, sex, body temperature, comorbidities and WBC, it was found that the use of IVIG was related to a decrease in the 28-day mortality rate. That finding was different from the results of our study. However, subgroup analysis in the study by Shao et al. showed that IVIG treatment was only able to significantly reduce the 28-day mortality rate in critically ill patients (defined by one of the following three criteria: a, respiratory failure requiring mechanical ventilation; b, shock; and c, multiple organ failure requiring ICU treatment). In severely ill patients (defined as having one of the following three conditions: a, respiratory rate ≥ 30 breaths/min; b, resting state SpO2 ≤ 90%; and c, PaO2/FiO2 ≤ 300 mmHg), the use of IVIG was not related to a decrease in the 28-day mortality rate. The severely ill patients included in the study by Shao et al. were similar to those included in our study, and the mortality rate was also similar to that in our study. In the study conducted by Xie et al. [16], severely ill patients in the IVIG group had a longer hospital stay than did those in the non-IVIG group [22.0 (18.0–30.0) vs 15.0 (13.0–22.0), *P* < 0.001]. That conclusion was consistent with the results of our study. The length of hospital stay was similar between the two studies.

IVIG has been used for the treatment of severe viral infections, bacterial infections and sepsis. One study has proved the clinical efficacy of IVIG [18], especially in viral infectious diseases. However, other studies have failed to confirm the clinical efficacy of IVIG [19], leading to a controversy regarding the application of IVIG in acute respiratory viral infectious diseases. Similar discrepancies have occurred among COVID-19 studies, possibly related to the severity of the disease, the timing and dosage of IVIG application, and the duration of the inflammatory response caused by the disease. An excessive inflammatory response is one of the main pathological changes in COVID-19 patients. A severe cytokine storm has been found to be related to increased mortality in severely ill patients. Like immunoglobulins, glucocorticoids are also used as nonspecific immunomodulatory drugs for adjuvant treatment of COVID-19 patients [20]. A large-scale randomized open label study in the United Kingdom showed that dexamethasone reduced the 28-day mortality rate in patients who received invasive mechanical ventilation (29.3% vs 41.4%; rate ratio, 0.64; 95% CI, 0.51 to 0.81) or patients who received oxygen therapy alone (23.3% vs 26.2%; rate ratio, 0.82; 95% CI, 0.72 to 0.94) [21]. However, dexamethasone was not beneficial to COVID-19 patients who did not require respiratory support (17.8% vs 14.0%; rate ratio, 1.19; 95% CI, 0.91 to 1.55). Findings from this study are similar to those found in other RCTs or in meta-analyses [22–24]. Subgroup analysis of Li's study showed that patients with critical illness and patients with ARDS were more likely to benefit from glucocorticoid administration [25]. This might be correlated with the inhibitory effect of glucocorticoids on inflammatory organ injury. However, in this study, severe COVID-19 patients were not found to have benefitted from IVIG, although IVIG, like glucocorticoids, is a nonspecific immunomodulatory agent.

This study had some limitations. First, this was a single-center retrospective cohort study of 113 patients that lacked sufficient representativeness and could not establish any causal relationship. Second, timing and doses of IVIG were not considered, although most patients were administered with 0.5 g/kg/d, recommend by Chinese guidelines. Third, various confounding factors that might have affected patient prognosis (such as PaO2/FiO2, troponin, and imaging changes) were not examined. Because of the small sample size of the cohort, we could not analyze which subgroup of patients, male vs female, high BMI vs low BMI, young vs old age, benefitted from immunoglobulin administration. Fourth, due to the clinical workload and situation at the time, data such as virus shedding time and immunoglobulin-related complications were not collected. In future, rigorously designed prospective studies should be performed to evaluate the role of IVIG in COVID-19.

## Conclusion

IVIG is not related to the in-hospital mortality rate and the use of mechanical ventilation in patients with severe COVID-19. These relationships need to be confirmed by prospective randomized controlled studies.

## Data Availability

All data supporting the conclusions of this article are included in this published article.

## Abbreviations

COVID-19: Coronavirus disease 2019
SARS-CoV-2: Severe acute respiratory syndrome coronavirus 2
IVIG: Intravenous immunoglobulin
COPD: Chronic obstructive pulmonary disease
WBC: White blood cell
LYM: Lymphocyte
PLT: Platelet
ALT: Alanine aminotransferase
AST: Aspartate aminotransferase
Cr: Serum creatinine
PT: Prothrombin time
DD: D-dimer
GC: Glucocorticoid
